# Occurrence of Vasospasm and Infarction in Relation to a Focal Monitoring Sensor in Patients after SAH: Placing a Bet when Placing a Probe?

**DOI:** 10.1371/journal.pone.0062754

**Published:** 2013-05-02

**Authors:** Christian T. Ulrich, Christian Fung, Hartmut Vatter, Matthias Setzer, Erdem Gueresir, Volker Seifert, Juergen Beck, Andreas Raabe

**Affiliations:** 1 Department of Neurosurgery, Bern University Hospital, Inselspital, Bern, Switzerland; 2 Department of Neurosurgery, Johann Wolfgang Goethe-University, Frankfurt/Main, Germany; University of Münster, Germany

## Abstract

**Introduction:**

Vasospastic brain infarction is a devastating complication of aneurysmal subarachnoid hemorrhage (SAH). Using a probe for invasive monitoring of brain tissue oxygenation or blood flow is highly focal and may miss the site of cerebral vasospasm (CVS). Probe placement is based on the assumption that the spasm will occur either at the dependent vessel territory of the parent artery of the ruptured aneurysm or at the artery exposed to the focal thick blood clot. We investigated the likelihood of a focal monitoring sensor being placed in vasospasm or infarction territory on a hypothetical basis.

**Methods:**

From our database we retrospectively selected consecutive SAH patients with angiographically proven (day 7–14) severe CVS (narrowing of vessel lumen >50%). Depending on the aneurysm location we applied a standard protocol of probe placement to detect the most probable site of severe CVS or infarction. We analyzed whether the placement was congruent with existing CVS/infarction.

**Results:**

We analyzed 100 patients after SAH caused by aneurysms located in the following locations: MCA (n = 14), ICA (n = 30), A1CA (n = 4), AcoA or A2CA (n = 33), and VBA (n = 19). Sensor location corresponded with CVS territory in 93% of MCA, 87% of ICA, 76% of AcoA or A2CA, but only 50% of A1CA and 42% of VBA aneurysms. The focal probe was located inside the infarction territory in 95% of ICA, 89% of MCA, 78% of ACoA or A2CA, 50% of A1CA and 23% of VBA aneurysms.

**Conclusion:**

The probability that a single focal probe will be situated in the territory of severe CVS and infarction varies. It seems to be reasonably accurate for MCA and ICA aneurysms, but not for ACA or VBA aneurysms.

## Introduction

The occurrence of symptomatic vasospasm in patients with aneurysmal subarachnoid hemorrhage (SAH) is a major complication [1]. Approximately 70% of patients develop an angiographic cerebral vasospasm (CVS) between days 3 and 14 after SAH [2]. Neurological deficits due to delayed cerebral ischemia or infarction occur in 20–40% of patients [1], [3]–[5]. The most potent risk factor for the development of CVS is the presence of thick clots in the basal cisterns [6]. Additional risk factors for development of a cerebral ischemia due to vasospasm are a poor clinical grade [7], acute and chronic hypertension [7], fever [7], intravascular volume depletion [8] and cigarette smoking [9]. Although pharmacological and hemodynamic rescue therapies are feasible [10], [11], often the timing of more aggressive treatments is problematic. Microdialysis, measurement of focal cerebral blood flow (CBF) or intraparenchymal tissue oxygen tension (ptiO2) are potentially sensitive and continuous techniques that could be used for diagnosis and treatment of hemodynamic consequences of CVS 12,13. These measurements, however, are highly focal and are most commonly beneficial when the site of sensor placement corresponds to the territory of the artery with the maximum vasospasm.

The widespread clinical assumption is that the maximum of CVS occurs at the vessel in contact with the largest clot that usually corresponds to the vessel harboring the ruptured aneurysm. However, data describing this correlation in the literature is limited [14], [15], [16] and therefore the rates of missing severe CVS or infarctions by monitoring with a focal sensor are unknown. The main issue of this study was to approve the generally accepted algorithm for choosing the side and site to place a focal sensor according to the vessel of aneurysm rupture based on our collective of SAH patients with angiographically severe CVS. We sought to quantify the likelihood that a focally placed probe would be situated in the territory where severe CVS or infarction occurs.

## Methods

### Database and Inclusion Criteria

Data for the study were collected from two similar databases established in both participating departments (Goethe University Hospital, Frankfurt/Main, Germany and Inselspital University Hospital, Bern, Switzerland). 100 consecutive patients with angiographically proven ruptured intradural aneurysms categorized as World Federation of Neurological Surgeons (WFNS) grade I to V and Fisher grade 3, and severe CVS between day 7 and 14 after hemorrhage were retrospectively selected. The CVS occurred in at least one major vessel territory (internal carotid artery [ICA], middle cerebral artery [MCA] M1, M2, anterior cerebral artery [ACA] A1, A2, or vertebrobasilary arteries [VBA]). CVS was regarded as severe when the narrowing of the arterial vessel lumen exceeded 50% of the normal caliber based on digital subtraction angiography (DSA) measurements.

The clinical diagnosis of CVS was made in patients who were suitable for neurological examination and had a newly diagnosed neurological deficit after exclusion of all other potential causes of neurological deterioration or a drop in Glasgow Coma Scale (GCS) of at least two points. Analgosedated and ventilated patients were monitored daily by transcranial Doppler sonography (TCD). Clinical signs of CVS or monitoring parameters triggered an angiography or stroke magnetic resonance imaging (MRI). In patients without clinical signs, these were performed routinely on day 7 after bleeding. All patients were treated according to a standardized protocol that has been described elsewhere [17].

Patient data was only available to the study leaders, and was anonymized for all others and for the current study. The study was registered with the ethics committees responsible for both university hospitals. There was no individual written patient consent because patients in the acute phase of SAH are often lethargic, confused, or in a coma. In general, a retroactive consent for analysis of data for patients with severe SAH is extremely difficult due to their generally poor condition, and the number of patients available to give retroactive consent is extremely limited.

### Protocol of Probe Placement and Analyses of Angiographic CVS

Side and site for sensor placement was determined using a standard algorithm according to the location of the aneurysm. Probe placement was as follows (see Table 1): for anterior cerebral artery (ACA) A1 segment (A1CA) aneurysms, the ipsilateral ACA territory was selected, and for anterior communicating artery (ACoA) or anterior cerebral artery A2 segment (A2CA) aneurysms, the right ACA territory was selected. For middle cerebral artery (MCA) and ICA aneurysms, the ipsilateral MCA territory was selected, and for VBA aneurysms, the right MCA territory was selected.

**Table 1:** Clinical algorithm used to guide placement of sensor relative to the location of the aneurysm.

Vessel territory of probe placement	Aneurysm bearing vessel
MCA right	MCA right, ICA right, posterior circulation
MCA left	MCA left, ICA left
ACA right	ACA right, AcoA
ACA left	ACA left

MCA = middle cerebral artery; ICA = internal carotid artery; ACA = anterior cerebral artery.

In the MCA territory, the sensor was inserted 10 mm in front of the coronary suture and 60 mm paramedian. For the ACA territory, the sensor was inserted 10 mm in front of the coronary suture and 20 mm paramedian. In both locations, the probe was placed subcortically at a depth of 20–30 mm below the dura mater level through a burr hole.

To determine whether severe CVS occurs in the vessel territory of ruptured aneurysm, 100 angiographies were analyzed. The presence of severe CVS was matched with the probe placement according to the described protocol. Therefore, our analytic procedure was purely theoretical.

Regardless of those conditions, the analysis of hypothetical probe placement in correlation to the site and side of severe CVS in the angiography was the same in patients with or without a cerebral probe. The cerebral probes were implanted in patients who were either in poor clinical condition (WFNS grade IV or V) that prevented neurological assessment (e.g., due to analgosedation and ventilation) with signs of CVS, or were in primarily good condition (WFNS I-III) with neurological deterioration due to CVS.

### Data Analyses

**Figure 1:**
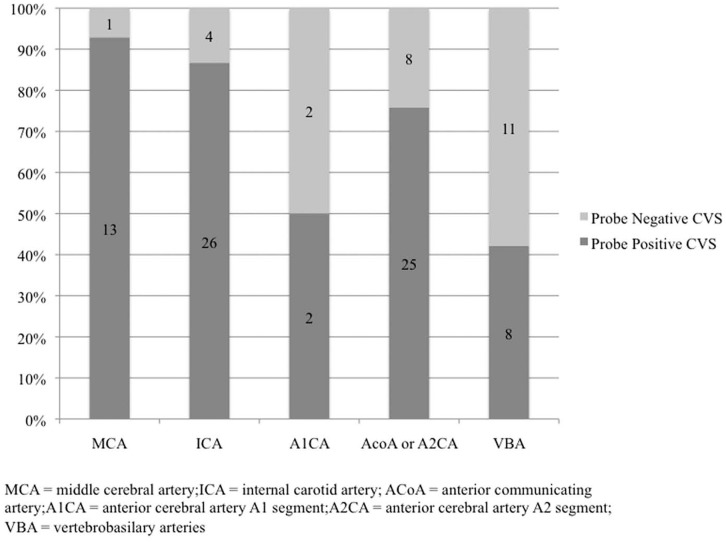
Percentage of patients, by aneurysm type, with corresponding position of probe in relation to CVS.

**Figure 2:**
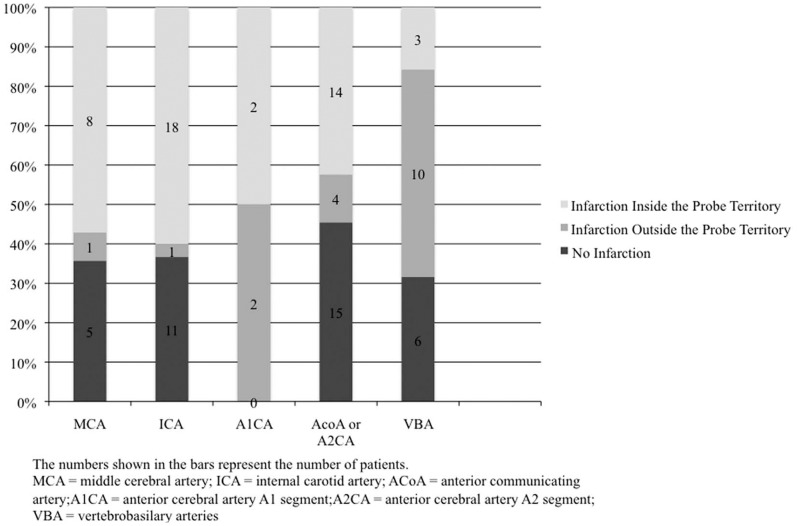
Percentage of patients, by aneurysm type, with probe in the territory of vessel infarction.

Data were recorded with IBM SPSS Statistics 12.0 (SPSS Inc., Chicago, IL). Descriptive statistics were used for analysis of the findings. The relation between aneurysm location and CVS, as well as between the CVS and cerebral infarction occurrence is given as percentage (%) in Figures 1 and 2.

We also analyzed the likelihood that the probe location as chosen by the described protocol corresponded to the territory of the vessels with severe angiographic vasospasm and/or infarction as documented in our database.

## Results

All patients included in this study were selected from the database because they had severe angiographic CVS (>day 6) of >50% arterial narrowing compared to the admission angiogram. We analyzed 14 MCA, 30 ICA, 33 AcoA or A2CA, 4 A1CA, and 19 VBA aneurysms. The median age was 47.7 years (range from 17 to 77 years). The male to female ratio was 1∶1.5. Clipping (65 patients) or coil embolization (35 patients) was performed within 72 hours of hemorrhage. Regardless of the angiographic analyses of severe CVS, 19 patients had an invasive cerebral probe (Licox®, Integra). The remaining 81 patients had no cerebral probe monitoring.

### Estimation of the Likelihood of Probe Placement in the Vessel Territory of Severe CVS

Severe CVS corresponded to the probe’s position (ipsilateral MCA territory) in 93% (n = 13) of MCA aneurysms, and in 87% (n = 26) of ICA aneurysms (Figure 1 and Table 2). The ipsilateral A1CA showed severe CVS in the probe area in 50% of A1CA aneurysms (n = 2). In AcoA and A2CA aneurysms where the probe was placed into the right ACA territory, CVS occurred in the probe’s territory in 76% (n = 25). In VBA aneurysms, severe vasospasm occurred only in 42% (n = 8) in the right MCA territory where the sensor was placed according to the protocol. Furthermore, severe CVS occurred outside the vessel territory of the probe in 7% of MCA aneurysms, 13% of ICA aneurysms, 24% of AcoA and A2CA aneurysms, 50% of A1CA aneurysms and 58% of VBA aneurysms (Figure 1 and Table 2).

**Table 2:** Number of patients according to aneurysm type, location of the probe and occurrence of infarction.

Aneurysm location	Total aneurysms	CVS in probe area	CVS outside probe area	Infarct insideprobe territory	Infarct outsideprobe territory	No infarct
MCA right	6	6	0	3	0	3
MCA left	8	7	1	5	1	2
ICA right	15	12	3	10	1	4
ICA left	15	14	1	8	0	7
A1CA right	3	1	2	1	2	0
A1CA left	1	1	0	1	0	0
AcoA, A2CA	33	25	8	14	4	15
VBA	19	8	11	3	10	6

MCA = middle cerebral artery; ICA = internal carotid artery; ACA = anterior cerebral artery; ACA = anterior cerebral artery; A1CA = anterior cerebral artery A1 segment, A2CA = anterior cerebral artery A2 segment: VBA = vertebrobasilary arteries.

We also analyzed whether a modified sensor placement algorithm might result in an improvement of sensitivity, but all other schemes resulted in a lower rate of diagnosing severe CVS.

### Frequency of Probe Location within the Territory of Vasospastic Infarction

As the main selection criteria for this investigation was acutely ruptured aneurysm and angiographically severe delayed vasospasm (>50% arterial narrowing), we also analyzed the correlation between the position of the probe and the likelihood of monitoring in a territory where vasospastic infarction occurs (Figure 2). In patients with ruptured MCA aneurysms (n = 14), infarction occurred in 64% of cases (n = 9). In 89% (n = 8) of patients with ruptured aneurysm and infarction, the infarction occurred in the probe’s location, i.e. the MCA territory ipsilateral to the MCA aneurysm. In one patient there was an infarction on the contralateral side and 5 patients had no infarctions. Of the patients with ICA aneurysms (n = 30), infarction occurred in 63% (n = 19). In 95% (18 out of 19 patients), infarction occurred ipsilaterally, i.e. within the probe’s location. Only one infarction occurred contralaterally and 11 patients had no infarction. Of the A1CA aneurysms (n = 4), infarction occurred in all 4 patients. In 50% of the cases, infarction occurred in the probe’s territory and 50% occurred outside the probe’s territory. Of the patients with AcoA or A2CA aneurysms (n = 33), infarction occurred in 55% of cases (n = 18). In 14 out of 18 patients, infarction occurred within the probe’s location and in 22% outside the probe’s location. Fifteen patients with AcoA or A2CA aneurysms had no infarction (46%). In patients with posterior circulation (VBA) aneurysms (n = 19), cerebral infarction occurred in 68% (n = 13). Of those, 23% of infarctions occurred within the territory of the probe (right MCA territory) and 77% outside the probe’s territory. In 6 patients with VBA aneurysms no infarction occurred (32%).

## Discussion

Monitoring focal cerebral blood flow or metabolic or tissue oxygen changes in an area that is considered at risk for ischemic injury after SAH may have an impact on treatment of CVS. The problem of highly focal measurements with these probes is well-known, but the numbers supporting this problem are limited [14], [15], [16]. Different methods of positioning a catheter into the vascular territory deemed at highest risk for developing CVS and hypoperfusion have been previously reported [12], [13], [18]. One study estimated about a 20% risk to misplace the probe and miss the area of hypoperfusion [12]. The insertion of two catheters for MCA and ICA aneurysms into the ipsilateral MCA and ACA territories, and ACoA aneurysms bilateral into the ACA area [13] was also described. However, these data indicated only a minor benefit for multiple probes compared with a proper unifocal monitoring approach [13].

According to the Consensus Meeting on Microdialysis in Neurointensive care, the positioning of catheters should be directed towards the tissue at risk, most likely the parent vessel territory [19]. However, the reliability of this procedure has not been evaluated. Sarrafzadeh et al. [18] proposed that the tissue at risk is most likely the parent vessel territory of the aneurysm. Recent investigations also have found a correlation between the distribution of CVS and ruptured aneurysm site [7], [20]–[22]. Harrod et al. [23] reviewed risk factors for CVS and found inconclusive circumstances for a correlation between ruptured aneurysm location and incidence of CVS, which would limit the value of focal measurements. The classical study by Fisher et al. [6] described the relation between a focal collection of blood as a risk factor for nearby CVS. Conversely, Graf and Nibbelink [24] found that 50% of patients with an ICA aneurysm, 45% of patients with a MCA aneurysm, and 35% of patients with an ACA aneurysm had localized or diffuse vasospasm. Furthermore, other studies have shown that the incidence of vasospasm is higher in patients with aneurysms of the medial circle of Willis, such as those of ICA, compared to patients with aneurysms located more peripherally [25], [26]. McGirt et al. [27] found that patients with ruptured posterior cerebral artery (PCA) aneurysms were 20-times less likely to develop symptomatic vasospasm. In contrast, other reports suggest that the frequency of vasospasm does not vary with anatomic position [28], [29]. For example, in a study of 100 consecutive patients with aneurysmal SAH, Bonilha et al. demonstrated no difference in the incidence of vasospasm as a function of aneurysm size or location [30]. Despite these findings, focal measurements are accepted as part of the clinical management in patients after SAH, especially to guide therapy when vasospastic hypoperfusion occurs. We therefore sought to quantify the risk of missing a severe vasospasm or infarction when using an accepted clinical algorithm to determine where to implant a focal sensor depending on the aneurysm location. Although cerebral ischemia after SAH also occurs without angiographically visible CVS of a large proximal artery [31]–[33], we found it necessary to select the patients for our study based on a morphological correlate.

For different aneurysm sites, we found different likelihoods of capturing severe CVS by a focal sensor. It was highest in MCA and ICA aneurysms, with a chance of 87–93% of a successful monitoring approach. In AcoA and A2CA aneurysms, the rate of successful monitoring was 76%, whereas in A1CA and VBA the rate was as low as 50% and 42% respectively, although this may be explained in part by the low number of A1CA cases. Aneurysms of the posterior circulation were monitored by placing a probe into the right MCA territory, which was in fact distant from the ruptured aneurysm. However, placement of a probe directly into the territory of the posterior circulation is not a routine clinical approach and appears to be more dangerous. On the other hand, also in VBA aneurysms, the thick diffuse or localized clot is also most often found around the circle of Willis.

According to the literature [1], [3], [5], [22], [33]–[35], vasospastic infarction may occur in up to 40% of patients or even up to 60% when MRI studies are used. In our study, the numbers of vasospastic infarctions were higher (63%) because we used some ‘enrichment’ strategies when selecting the patients for this analysis that were shown to increase the rate of vasospasm and infarction [36]. As our primary goal was to investigate the likelihood that a localized sensor would pick up the vasospasm or infarction, we used the criteria of ‘WFNS grade IV’, ‘Fisher grade 3’ [6], and angiographically proven severe cerebral vasospasm. This selection strategy is likely to explain the high rate of infarction in our study.

By placing the cerebral probe into the vessel territory of corresponding ruptured aneurysm we observed, to some extent, a reliable monitoring approach. This was particularly reliable for MCA, ICA, and ACoA aneurysms. Concerning the ACA and VBA aneurysms, the CVS location is inconsistent due to the previously mentioned reasons. Despite the study limitations, we believe that our results support the use of these methods for the management of probe placement for specific aneurysm types.

### Conclusions

The probability that a single focal probe will be situated in the territory of severe CVS and infarction varies over a wide range. More reliable CVS or infarction detection was observed in MCA and ICA aneurysms compared to A1CA and VBA aneurysms. In our opinion, focal ptiO2 or CBF or microdialysis measurements are useful for MCA and ICA aneurysms, but may have a high (25–50%) failure rate in patients with VBA and ACA aneurysms.
